# Idiopathic Spontaneous Pneumomediastinum in an Adolescent

**DOI:** 10.7759/cureus.17941

**Published:** 2021-09-13

**Authors:** Hamza Malik, Sophie Boyes, Lee Dobson, William Kent

**Affiliations:** 1 Emergency Department, Royal Devon and Exeter NHS Foundation Trust, Exeter, GBR; 2 Respiratory Medicine, Royal Devon and Exeter NHS Foundation Trust, Exeter, GBR

**Keywords:** spontaneous, idiopathic, adolescent and young adults, emergency department, pneumomediastinum

## Abstract

Idiopathic spontaneous pneumomediastinum (ISPM) is a diagnosis of exclusion after a spontaneous pneumomediastinum (SPM) occurs without any identifiable predisposing factors or known aetiology. It is a rare diagnosis in adolescents, with a few cases reported in the literature. To increase awareness of this rare diagnosis, we present a case of a 17-year-old, fit and healthy male who presented with acute atraumatic chest pain. On examination, surgical emphysema in the supraclavicular fossa was identified. His chest X-ray and a subsequent computed tomography (CT) of the thorax showed extensive pneumomediastinum, with infiltration of air into the soft tissues of the neck and upper arms, but with no identifiable cause. On follow-up, he remained asymptomatic, and a repeat CT of the thorax four weeks after his initial presentation showed complete resolution of the pneumomediastinum. Once confirmed, ISPM is expected to resolve spontaneously without complications, with a very low rate of recurrence in nearly all cases.

## Introduction

Idiopathic spontaneous pneumomediastinum (ISPM) is a diagnosis of exclusion defined as spontaneous pneumomediastinum (SPM), without any precipitating factors or identifiable aetiology after investigations [1,2]. SPM itself is an unusual diagnosis in adolescents, with an estimated incidence of only 1 in 800 to 1 in 42,000 [3], and ISPM makes just a small proportion of these cases [4,5]. We believe that our presentation is a case of genuine ISPM in an adolescent without any pre-existing medical conditions or any predisposing or precipitating factors, and ISPM has not been reported at this age.

## Case presentation

Summary

The patient was a 17-year-old tall thin male who attended the Emergency Department (ED) late one evening reporting chest pain. The patient described the sudden onset of pain at 18:00 that day and characterised it as a sharp, non-radiating pain that worsened on inspiration. The pain severity was 7/10, which was relieved by oral morphine and had no obvious precipitant. He denied any overt shortness of breath but reported a ‘bubbly’ feeling in his supraclavicular fossa. The patient reported that he had felt a bit ‘funny’ in his head and chest earlier in the day but had otherwise been well with no trauma, vomiting or respiratory symptoms. The patient had no significant past medical history or surgical interventions. He was a non-smoker and had minimal alcohol intake. He had no significant familial history, in particular no history of connective tissue disorders or sudden death; his mother confirmed this as well.

Investigations

**Figure 1:**
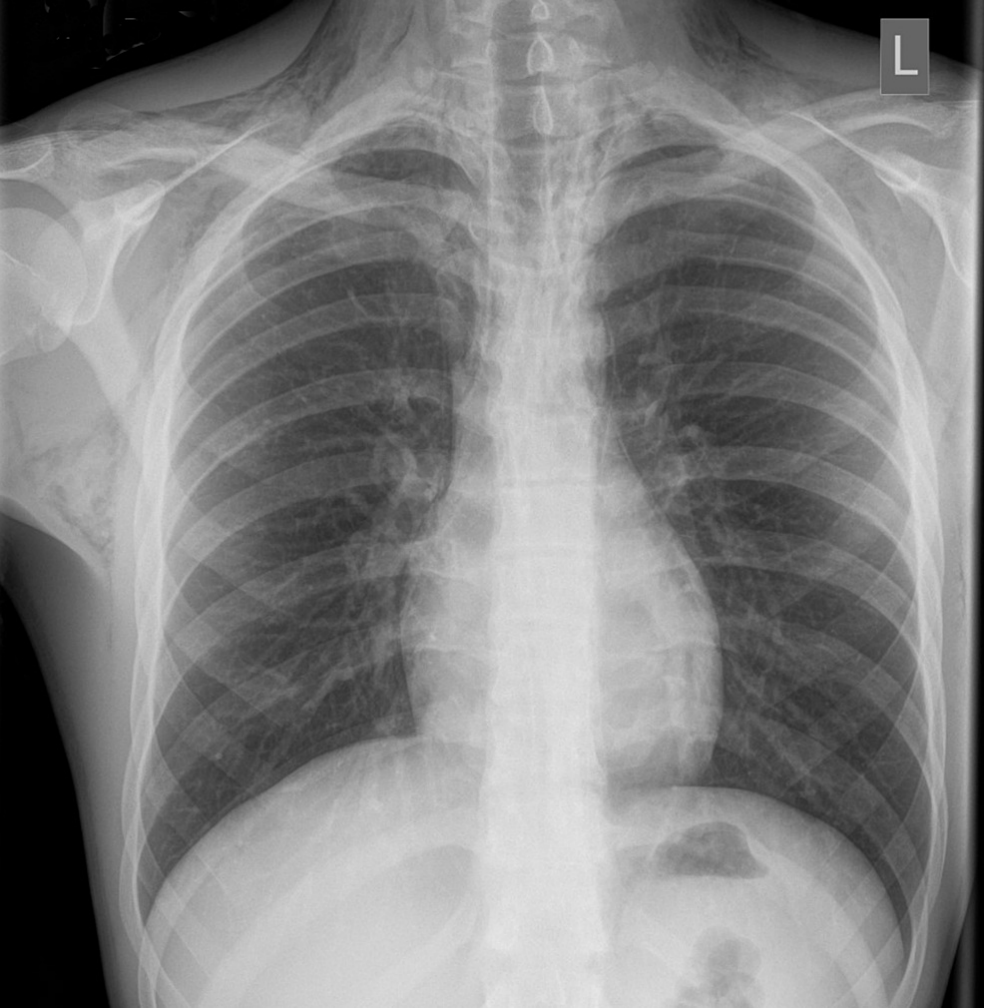
Chest X-ray on day 1 showing neck soft tissue emphysema and pneumomediastinum.

On examination, the patient appeared comfortable. His airway was patent, trachea was central and he had clear chest sounds with equal inspiratory effort. He had obvious surgical emphysema in his supraclavicular fossa but no other signs of chest wall injury. His observations were all within normal range. Routine blood tests were unremarkable. The initial investigation performed was a chest X-ray (Figure 1), which showed significant surgical emphysema bilaterally in the chest wall and soft tissues of the neck as well as some free air around the mediastinum and cardiac border, indicating a pneumomediastinum.

The limitations of the chest X-ray are that due to a single view; it was difficult to assess the full extent of free air in the chest wall and surrounding mediastinum. Additionally, the relatively poor sensitivity of this imaging meant that we were unable to determine a cause for the pneumomediastinum.

**Figure 2:**
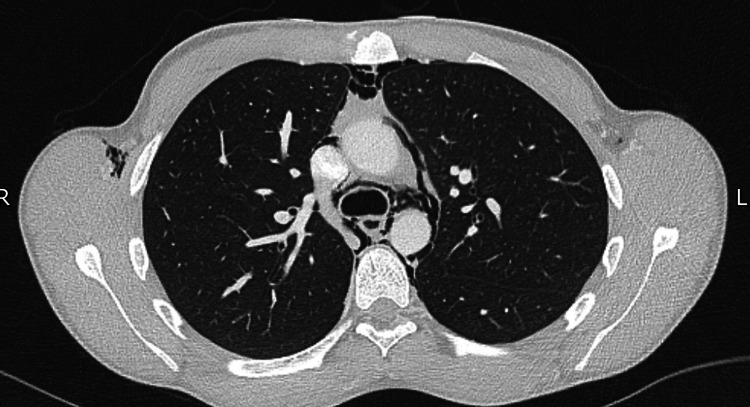
CT of the thorax image on day 1 showing pneumomediastinum.

The next investigation was a CT of the thorax (Figure 2) to assess for the source of the air leak and more accurately visualise the distribution of free air in the thorax. The CT also showed extensive pneumomediastinum and described extension into the neck and upper arms. Although the CT allowed a clearer view of the distribution of free air, the aetiology remained elusive. The CT scan showed no evidence of any changes with the pulmonary parenchyma or pleura that might have contributed to a pneumomediastinum. The patient was then admitted to the hospital and seen by the paediatric, respiratory and surgical teams. A barium swallow test was performed the following morning, which showed no evidence of oesophageal contrast leak with normal passage of contrast into the stomach.

Differential diagnosis

Pneumomediastinum secondary to chest trauma was initially thought to be the most likely diagnosis as there was no history of underlying medical conditions which may have predisposed him to a pneumomediastinum. The patient unequivocally denied any history of this, and because of the lack of evidence of trauma on CT imaging, it could be reasonably excluded.

Pneumomediastinum secondary to oesophageal perforation, aside from iatrogenic causes, can be the result of Boerhaave’s syndrome due to excessive vomiting or straining. In this case, the patient again denied any history of such precipitants, and the barium swallow examination confirmed an intact oesophagus. ISPM, a diagnosis of exclusion, could only be made once other causes had been investigated and ultimately ruled out.

Treatment

**Figure 3:**
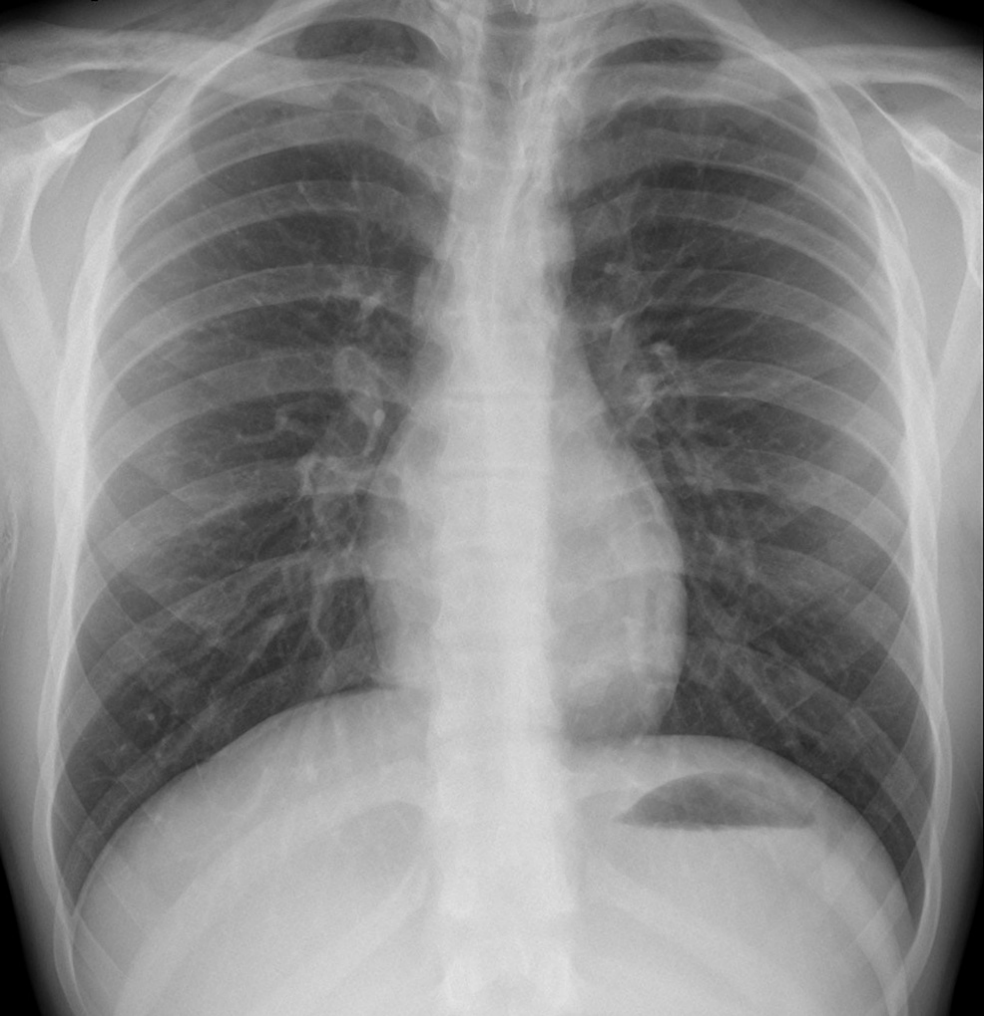
Chest X-ray on day 3 showing partial resolution of emphysema and pneumomediastinum.

As the patient was only 17 years old, he was referred to the General Surgery, Paediatric and Respiratory teams for joint care. The most important aspect of managing this case was to identify and correct any cause for the pneumomediastinum. As no cause was found in this case, the patient was managed conservatively guided by a thoracic medicine consultant. He was observed in the paediatric ward for 24-48 hours and underwent a further erect chest X-ray (Figure 3) after his initial X-ray, which showed a reduction in the volume of the free air within the supraclavicular fossa and the mediastinum.

Outcome and follow-up

**Figure 4:**
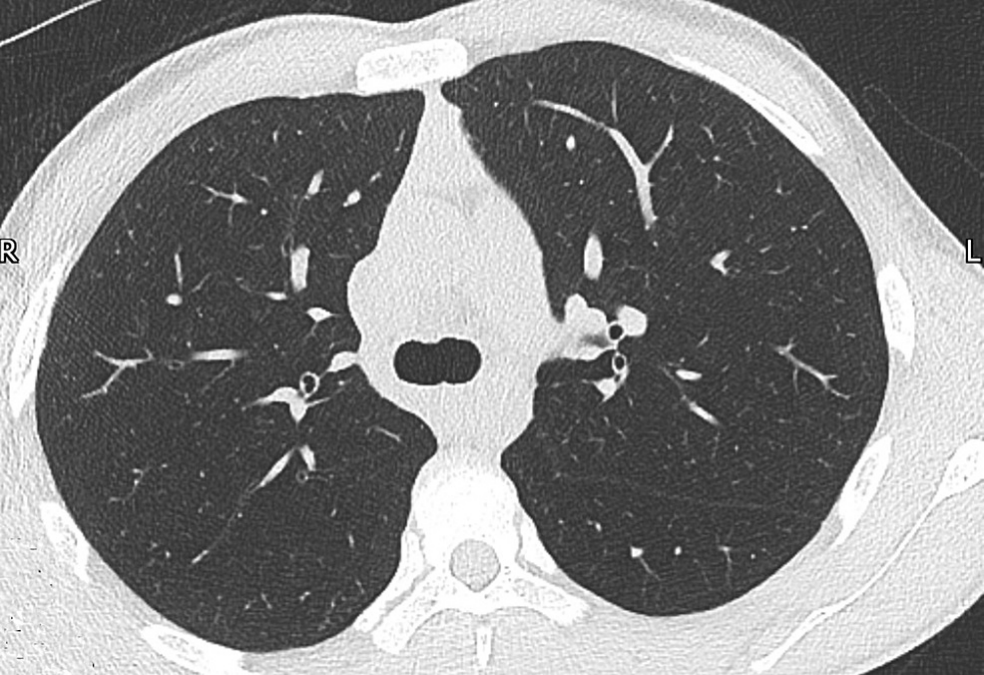
CT of the thorax image after four weeks showing resolution of pneumomediastinum.

After a short period of observation, the patient was discharged 24-48 hours after admission. He had clinically improved within 24 hours and reported that his chest pain had resolved. The surgical emphysema had reduced as well. The patient was seen for a follow-up CT of the thorax at four weeks (Figure 4) following this episode to ensure resolution of his pneumomediastinum. As his four-week investigations were normal and he was asymptomatic, no further follow-up was required.

## Discussion

ISPM refers to SPM without triggering factors, known lung disease or underlying aetiology contributing to it [6]. It is usually a benign condition and treated conservatively [5]. We presented a case of ISPM, highlighting the lack of guidelines or consensus regarding the definition and management of ISPM.

There are many case reports and a few literature reviews which highlight SPM in children and adolescents. However, there are hardly any cases that are reported as ISPM and they seem to be mentioned under the umbrella term of SPM. A retrospective study including 53 children found that 15 % of SPM cases were ISPM cases [7]. Another study from Taiwan found ISPM to be 50% of SPM cases [8]. Chidambaram and Donekal described SPM and subcutaneous emphysema without any predisposing factors or trauma in a 14-year-old male, who recovered with conservative treatment [9]. They did not classify or report this case as ISPM. Three studies present SPM in adolescents, without any triggering factor or underlying aetiology, with good recovery after conservative treatment [10-12]. They also did not use the term ISPM for these cases. Nounla et al. reported SPM in a three-year-old and a 15-year-old [5]. The three-year-old had dyspnoea two days ago but presented with a fever and signs of infection. A tension pneumomediastinum developed in her case requiring treatment with respiratory support, antibiotics and collar mediastinotomy. A CT confirmed pneumomediastinum, but all other investigations could not reveal a source or a trigger factor. She was discharged home after 12 days of admission. The 15-year male developed chest pain and dyspnoea while playing football. Chest X-ray confirmed SPM, while the rest of his investigations were normal. He was treated conservatively with the usual follow-up three months later. No other cases have been reported as ISPM in adolescents according to the best of our knowledge.

ISPM is considered to be a benign condition [5,6], but there is a lack of evidence regarding its management. There are no robust guidelines to investigate and treat ISPM in adolescents. There is a possibility that follow-up chest X-ray or CT of the thorax may not be required in asymptomatic patients with ISPM, given its benign course and risks due to radiation exposure in this age group. A consensus is required to define ISPM and set guidelines to manage it.

## Conclusions

SPM is a rare diagnosis, and very few cases have been reported in adolescents. Initial history, examination, blood tests, chest X-ray, CT of the thorax and barium swallow can lead to a diagnosis of ISPM by exclusion. There are no current guidelines to manage ISPM in adolescents. We presented the case of an adolescent with true ISPM, who recovered after conservative management. We suggest that once ISPM has been diagnosed, follow-up investigations might not be required in young asymptomatic patients.

## Appendices

Patient’s perspective

During a late supper I was aware of some chest pain and that my neck felt odd - almost like there was bubble-wrap under my skin. I had never experienced either of these things before. I decided to go to bed, but the chest pain was getting worse, and I discovered it was too painful to lie down on my back. My Mum called 111 and they said I should come to A&E. I had some paracetamol, but this did not really relieve the pain and after a few hours it was almost unbearable. Around 2.30am, a dose of morphine finally eased some of the pain. The bubbles of air could be felt around my neck and shoulders, and the X-ray showed air in other cavities of my torso. Gradually during Monday, the pain and bubbles reduced, and by Tuesday I was starting to feel better, although was very hungry as I had not been allowed to eat! It did seem to be a mystery why and how this had happened, as there was no obvious 'hole' though which the air had escaped into my body. With some food and continued rest, by Wednesday I felt OK and did not need any painkillers and was able to come home. I was told to not play the saxophone until the follow-up CT scan and to not exercise too vigorously. The results of that scan were all clear and I have been fine since, although did feel some puffiness in my neck after an active weekend in June 2021. There was no pain and it quickly cleared and so we did not seek medical help.
